# Utilisation review of thromboelastography in intensive care

**DOI:** 10.1186/cc14424

**Published:** 2015-03-16

**Authors:** J Aron, A Gibbon, C Ward, J Ball

**Affiliations:** 1 St George's Hospital, London, UK

## Introduction

This review aims to assess the value of thromboelastography (TEG) on the general ICU, which has not been previously demonstrated. TEG is a near-patient assessment of whole blood coagulation and fibrinolysis, which reduces transfusion requirements during cardiothoracic surgery and liver transplantation.

## Methods

A prospective audit of TEG tests performed on patients being treated on a general ICU was conducted over 2 months.

## Results

**Figure 1:**
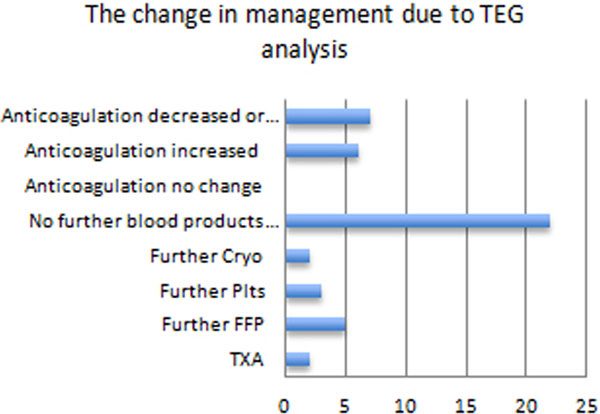
**Change in management due to TEG results**.

A total of 332 TEG tests were performed with a failure rate of 29.8%. Seventy-eight audit sheets were collected. Mean patient age was 68.9 years and mean APACHE II score was 18.1. Admissions included trauma (33.0%), perioperative (43.6%), haemorrhage (42.3%) and sepsis (21%). Standard tests of coagulation demonstrated 22 deficits in coagulation which were not identified as functionally significant with TEG. Of these, 20 had abnormal clotting factor activity as measured by the INR/APTTr and 13 patients were thrombocytopenic. In total, 52.6% documented that the TEG result changed the management of the patient. In 46.8% of these cases no further blood products were required. In 41% there was no documentation. See Table 1 and Figure 1.

**Table 1:** Summary of concordance with standard tests versus TEG analysis.

	Both abnormal	TEG normal/standard abnormal	Standard normal/ TEG abnormal
Clot factor deficit	9 (11.5)	20 (25.6)	3 (3.8)
Platelet deficit	11 (14.1)	13 (16.6)	2 (2.6)
Fibrinogen deficit	5 (6.4)	4 (5.1)	5 (6.4)

Data presented as *n *(%).

## Conclusion

TEG analysis suggested that 22 patients who were identified as coagulopathic with traditional measures of coagulation did not have a functional deficiency. Over one-half of TEG studies resulted in a change in management and in 46.8% no further transfusions were required. There was a high technical failure rate and a low audit return rate, which may indicate the need for further training.
